# Plasticity of Sorghum Stem Biomass Accumulation in Response to Water Deficit: A Multiscale Analysis from Internode Tissue to Plant Level

**DOI:** 10.3389/fpls.2017.01516

**Published:** 2017-09-01

**Authors:** Lisa Perrier, Lauriane Rouan, Sylvie Jaffuel, Anne Clément-Vidal, Sandrine Roques, Armelle Soutiras, Christelle Baptiste, Denis Bastianelli, Denis Fabre, Cécile Dubois, David Pot, Delphine Luquet

**Affiliations:** ^1^CIRAD, UMR AGAP Montpellier, France; ^2^CIRAD, UMR SELMET Montpellier, France

**Keywords:** sorghum, stem biomass, water deficit, internode growth, (non)-structural carbohydrates, lignocellulose, cell-wall, tissue histology

## Abstract

Sorghum is increasingly used as a biomass crop worldwide. Its genetic diversity provides a large range of stem biochemical composition suitable for various end-uses as bioenergy or forage. Its drought tolerance enables it to reasonably sustain biomass production under water limited conditions. However, drought effect on the accumulation of sorghum stem biomass remains poorly understood which limits progress in crop improvement and management. This study aimed at identifying the morphological, biochemical and histological traits underlying biomass accumulation in the sorghum stem and its plasticity in response to water deficit. Two hybrids (G1, G4) different in stem biochemical composition (G4, more lignified, less sweet) were evaluated during 2 years in the field in Southern France, under two water treatments differentiated during stem elongation (irrigated; 1 month dry-down until an average soil water deficit of -8.85 bars). Plant phenology was observed weekly. At the end of the water treatment and at final harvest, plant height, stem and leaf dry-weight and the size, biochemical composition and tissue histology of internodes at 2–4 positions along the stem were measured. Stem biomass accumulation was significantly reduced by drought (in average 42% at the end of the dry-down). This was due to the reduction of the length, but not diameter, of the internodes expanded during water deficit. These internodes had more soluble sugar but lower lignin and cellulose contents. This was associated with a decrease of the areal proportion of lignified cell wall in internode outer zone whereas the areal proportion of this zone was not affected. All internodes for a given genotype and environment followed a common histochemical dynamics. Hemicellulose content and the areal proportion of inner vs. outer internode tissues were set up early during internode growth and were not drought responsive. G4 exhibited a higher drought sensitivity than G1 for plant height only. At final harvest, the stem dry weight was only 18% lower in water deficit (re-watered) compared to well-watered treatment and internodes growing during re-watering were similar to those on the well-watered plants. These results are being valorized to refine the phenotyping of sorghum diversity panels and breeding populations.

## Introduction

The objective of producing bio-based energy and industrial products must be adjusted to achieve required biomass production while meeting food and feed demand. Biomass crops therefore expected to be primarily cultivated on marginal lands, generally characterized by heterogeneous and shallow soils prone to the occurrence of water deficit (Rooney et al., 2007; Anami et al., 2015). In this respect, sorghum is a suitable crop and stem biomass production became in the last decade a target of sorghum breeding programs (Almodares and Hadi, 2009; Anami et al., 2015). Sorghum is the 5th cereal crop worldwide for grain production but can also produce large amounts of vegetative biomass in a 3–4-month cropping season particularly under temperate climates [more than 30 t.ha^-1^ in Italy (Garofalo and Rinaldi, 2011) and France (Trouche et al., 2014)]. It is characterized large genetic diversity for stem biochemical and physical properties, of high value for diverse end- products (Richie and McBee, 1991; Mace et al., 2013; Trouche et al., 2014). In addition, sorghum is drought tolerant and water use efficient (Zegada-Lizarazu and Monti, 2012; Borrell et al., 2014), which makes it adapted for water saving practices or water-limited cropping situations as met in marginal lands (Berenguer and Faci, 2001; Vasilakoglou et al., 2011; Anami et al., 2015). This is becoming more challenging with climate change that will increase the frequency of dry spells in sorghum cropping areas (Srivastava et al., 2010; Fu et al., 2016; Potgieter et al., 2016).

The environmental and physiological control of grain sorghum production and its drought response is well studied and implications for crop improvement were defined (Vadez et al., 2011; Borrell et al., 2014). The genetic control of sorghum grain production (Tuinstra et al., 1997; De Alencar Figueiredo et al., 2010) and its post-flowering drought tolerance (leaf stay-green: Crasta et al., 1999; water use efficiency: Dugas et al., 2011; Fracasso et al., 2016) were explored. Recent studies aimed to dissect genetic architecture and variability of stem biomass production and composition (sweet sorghum: Murray et al., 2009; Anami et al., 2015; biomass sorghum: Murray et al., 2008; Salas Fernandez et al., 2009; Trouche et al., 2014; Turner et al., 2016). These studies reported contradictory relationships between biomass production and composition that were only partly due to genotype. This was possibly due to the fact that they relied on the phenotyping of complex production and quality traits, in different genetic backgrounds and environmental conditions without considering the component traits at which the genotype × environment interactions (GxE) could be explained. Moreover, none of these studies dealt with drought effect. The depressive effect of water deficit on cereal internode length was reported in several studies (e.g., sweet sorghum: Tsuchihashi and Goto, 2005; maize: Bennouna et al., 2004) but these studies did not report internode biomass composition. Almodares et al. (2013) and Tovignan et al. (2016) (at whole stem level) and Ghate et al. (2017) (at internode level) reported an increase in soluble sugar accumulation in sweet sorghum stem under a post-flowering water deficit, i.e., when internode growth was completed. Zegada-Lizarazu and Monti (2013) confirmed this result at stem level and for one sorghum genotype in response to a water deficit applied before flowering; interestingly, a reduction of stem biomass accumulation and structural carbohydrate content was reported under these conditions.

The GxE underlying the production and the composition of sorghum stem biomass and their relationship with water deficit are thus poorly understood, as the component traits that cause phenotypic plasticity are not well identified. Several studies suggested that the phenotypic plasticity of biomass production and composition in response to environmental conditions can be explained at organ (internode) level (Gutjahr et al., 2013; Ghate et al., 2017 for sugar content response to photoperiod and drought respectively; Jung, 2003; Matos et al., 2013 for cell-wall deposition). To our knowledge, no study addressed drought response of internode growth, soluble sugar accumulation and cell wall lignocellulose. Jung and Casler (2006) reported that lignin deposition in the cell wall of the internode happens earlier and faster in the sclerenchyma and outer parenchyma compared to the internal zone of the internode. This suggests that not only the organ but also the tissue level should be studied to understand the phenotypic plasticity of stem traits. A dedicated methodology was recently reported for the study of sorghum stem structural traits (Jaffuel et al., 2016).

The present study aims at identifying the morphological, biochemical and histological traits participating in internode elongation and stem biomass accumulation and their response to water regime, at tissue, organ and plant level. For this purpose, two biomass sorghum hybrids, with similar biomass production potential and phenology but different soluble sugar and lignocellulosic contents (traits of economic interest), were evaluated in the field in two consecutive years in Southern France, under two contrasted water treatments. The results are discussed in view of refining phenotyping and modeling of biomass sorghum and biomass crops in general.

## Materials and Methods

### Plant Material

Two biomass sorghum hybrids were studied: Biomass 140 (RE1xAE1, from EUROSORGHO, France) and RE1xAR4, named respectively G1 and G4 thereafter. These two hybrids have a common male parent (RE1) with characteristics for biomass production (stem tallness, vigor, and long cycle) and two different female parents (AE1 and AR4 respectively). The female parents have similar phenology, but different internode composition: AE1 is more digestible and AR4 is sweeter (according to CIRAD breeders and breeding partners). The two hybrids had similar numbers of days to flowering; 111 vs. 116 in 2013 and 105 vs. 110 in 2014 for G1 and G4 respectively). Hybrids were chosen instead of lines because of greater vigor and biomass production. The choice of two hybrids with different biochemical composition served to study possible association of histochemical traits with phenotypic plasticity in response to drought. Similar phenology was essential in order to compare the effect of water deficit during the same developmental phase, namely stem elongation. Seeds were provided by CIRAD, RAGT2n^1^ and EUROSORGHO^2^.

### Experimental Details

The two hybrids were sown on the DIAPHEN field phenotyping platform in Mauguio (South of France^3^; Delalande et al., 2015; 43°36′43″N, 3°58′20″E) during the summer seasons 2013 and 2014 (sowing on May 22nd and May 23rd respectively). A different field was used in 2013 and 2014 but both had similar soil type, representative of DIAPHEN site (see website for details). Plants were grown in open field with two water treatments: Well-Watered (WW), the water being supplied with a mobile ramp of sprinklers; and Water Deficit (WD) (**Table 1**). Irrigation consisted of a 10 mm water supply two times per week. WD consisted of a 1-month dry-down period that began when plants had, on average, 11 ligulated (expanded) leaves on the main stem. The stage of 11 ligulated leaves was chosen as it corresponds to the onset of rapid elongation of internodes (Gutjahr et al., 2013; Perrier L, unpublished data). The experiment had a randomized complete block design with 4 and 3 replications in 2013 and 2014, respectively. The individual plot had 7 m long rows spaced at 0.8 m (8 and 4 rows per plot respectively in 2013 and 2014). 18 seeds were sown per linear meter. The two water treatments were separated by a band of bare soil of 20 m to avoid any hydrological communication between treatments.

**Table 1:** Meteorological comparison between years, treatments (WW: well-watered and WD: water deficit) and stages (beginning of stress, end of stress and harvest time) for cumulated thermal time (°Cd), radiation (PAR in J.cm^-2^), rainfall (mm) and water supply (irrigation and rainfall, mm).

		Initiation of stress		End of stress		Harvest	
				
		05/07/2013	08/07/2014	01/08/2013	31/07/2014	26/09/2013	30/09/2014
Cumulated thermal time (°Cd)		370.2	487.3	732.4	785.6	1395.5	1444.5
Cumulated PAR (MJ.m^-2^)		521.88	509.24	826.66	750.06	1355.70	1272.85
Cumulated rainfall (mm)		43.2	45.4	47.0	58.2	75.2	363.4
Cumulated water supply (mm)	*WW*	138.2	140	211	275.7	368.2	735.5
	*WD*	138.2	143.1	160	188.9	317.2	683


The predawn leaf water potential was measured at 4AM in the field during the stress period using a pressure chamber (PMS-1000, Corvallis, OR, United States) on three plants per block and genotype in the WD treatment and in 2014 only. The WD blocks presented on average a predawn leaf water potential of -8.85 bars at the end of the dry-down period when internodes were sampled for histochemical analyses described below. There was no significant difference between blocks and genotypes. The mean daily temperature measured at two meters varied between 13°C and 27°C and the mean daily PAR (Photosynthetically Active Radiation) between 5.5 and 13.60 MJ.m^-2^.d^-1^ during the growing period. The thermal time was computed from sowing time by cumulating daily average temperature reduced by base temperature, considered at 11°C for sorghum (Kim et al., 2010) (**Table 1**).

#### Non-destructive Measurements of Plant Phenology and Growth

The numbers of ligulated and green leaves on the main stem were counted and the plant height [PHT (cm), from the soil to the ligule of the youngest ligulated leaf] were measured on three plants per plot every week in 2013 and every 2 weeks in 2014. These plants were also used to estimate the number of days from emergence to flowering and were harvested during grain filling. In addition, in 2014, they were used at final harvest for measuring the length and diameter of all elongated internodes of the main stem. In 2013, only the internodes sampled for biochemical and histological analyses (as described below) were characterized for their length and diameter (two per sampling dates and per plant). Tillering was almost nil and the few tillers that appeared died before flowering.

#### Biomass Measurements

Three plants per plot were sampled at two stages: the end of the stress period (plants in the WW treatment with 17 ligulated leaves on average on the main stem), and final harvest. The stem, green leaves and panicle were separated and pooled for the three plants sampled within a plot. Total fresh weight was measured for each organ type. A sub-sample of each was dried at 60°C during 72 h in an oven and used to calculate water content. Based on water content and total fresh weight, the dry weight of each organ type per plant was computed.

#### Internode Histochemical Analyses

In each plot, nine plants were sampled to perform analyses at internode level. Three plants were used for histological analyses, two for wet biochemical analyses and four for NIRS (Near InfraRed Spectrometry) predictions.

In 2013, two internode ranks were sampled (second and fourth internodes below the youngest ligulated leaf phytomer) at five dates (two during the stress period, one at the end of stress, one 3 weeks after the stress period and one at final harvest stage, i.e., 663°Cd after the end of the stress period). In 2014, four internode ranks were sampled (internode from the last ligulated leaf phytomer, second, fourth and sixth internodes below the last ligulated leaf phytomer) at three stages (end and 3 weeks after the end of the stress period and harvest, i.e., 659°Cd after the end of the stress period). All sampled internodes in the WW treatment were analyzed for biochemical and histological traits. This was used to evaluate the dynamic patterns of each variable along internode positions and plant age. In the WD treatment, only two internode ranks were considered (2nd and 4th below the last ligulated leaf) at common sampling stages between the 2 years: end of the stress period and harvest stage. The ranks of sampled internodes were thus different between the two sampling stages in the WD treatment.

##### Histological analyses

**FIGURE 1:**
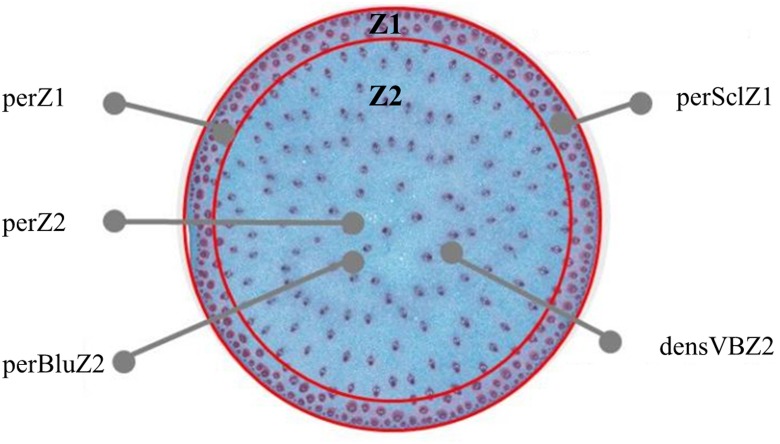
Cross section of sorghum internode, with identification of an outer (Z1) and a central (Z2) zone. Z1 is characterized by its area in % of internode section area (perZ1), the percentage of sclerenchyma tissue (red stained) in % of Z1 area (perSclZ1); Z2 is characterized by its area in % of internode section area, the percentage of blue tissue in percentage of Z2 area (perBluZ2), the density of vascular bundles in Z2 in number of vascular bundles per mm^2^ (densVBZ2). Example of G1 (Biomass140) in the well-watered treatment in 2013. Coloration by Fasga.

A 1 cm long segment was cut in the median part of each sampled internode. It was fixed in a buffer made of phosphate 100mM (pH 7.2), with 1% (v/v) glutaraldehyde and 2% (v/v) (w/v) caffeine during 48 h at ambient temperature and then conserved in 60% ethanol. The stem segments were then cut in thin slices of 90 μm using a *Vibratome* (Microm HM 650 V). The slices cut with the best precision were thereafter stained overnight using a Safranin and Alcian blue solution (Fasga; Tolivia and Tolivia, 1987) diluted at 1/7. The staining solution consisted of 14 ml of Alcian blue (0.5% in ethanol), 2 ml of Safranin O (1% in water), 1 ml of acetic acid, 30 ml of glycerin, and 19.5 ml of distilled water. After staining, the sections were rinsed twice during 5 min with distilled water and mounted on glass slides in glycerol (50/50). The Fasga staining colored lignified tissues red, whereas non-lignified or poorly lignified tissues were colored blue. Glass slides were then scanned with a *Nanozoomer* Hamamatsu and converted to high resolution images. Images were analyzed with the open-source ImageJ freeware^4^ (Ferreira and Rasband, 2010) and a dedicated script to quantify the following traits (**Figure 1**): the outer zone (Z1) area in % of internode section area (perZ1), the percentage of sclerenchyma tissue (red stained) in Z1 in % of Z1 area (perSclZ1), the percentage of blue tissue in the central zone of the internode (Z2) in % of Z2 area (perBluZ2) and the density of vascular bundles in Z2 (number per mm^2^, densVBZ2). Z1 and Z2 were delimited visually based on the anatomical difference between the two zones (**Figure 1**).

##### Biochemical composition analysis

The lignin content was quantified by two different methods, a gravimetric method quantifying acid detergent lignin (ADL) and a spectroscopic method with acetyl bromide as reagent (AcBL). These two determinations are complementary to describe lignin content (Fukushima et al., 2015).

The predictions of lignin, cellulose and hemicellulose contents were derived from NIRS based on the Van Soest reference method (Van Soest et al., 1991). This method provides estimates of total fiber (NDF, neutral detergent fiber, expressed in percentage of dry matter, %DW), lignocellulose (ADF, acid detergent fiber, expressed in %DW) and lignin (ADL, acid detergent lignin, expressed in %DW). The internodes of a given rank below the last ligulated leaf sampled on 4 individual plants per plot were pooled and dried during 72 h at 60°C. The dried samples were ground at a 1 mm sieving size and NIR spectra were acquired with a NIR system 6500 spectrometer (FOSS NirSystem, Laurel, MD, United States).

The calibration models trained on internode chemical traits were based on 660 internode samples including different growth stages and internode positions. ADF, NDF and ADL were used to calculate two additional traits: Hemi_VS (hemicellulose content computed as NDF-ADF, %DW) and Cell_VS (cellulose content computed as ADF-ADL, %DW).

The protocol for lignin determination by acetyl bromide (AcBL) was adapted from Fukushima and Hatfield (2001). Internode samples were lyophilized after sampling and crushed (<100 μm) with mixer mill (Retsch MM301). Cell wall residues (CWR) were prepared from 100 mg of the powder. Samples were washed twice in 5 ml of distilled water at 80°C. After centrifuging (10 min, 10000 rpm), the pellet was rinsed twice in 5 ml of absolute ethanol for 15 min at 80°C, then rinsed twice in 5 ml of acetone at room temperature for 10 min and left to dry under a fume hood overnight at room temperature. The CWR was weighed to calculate the percentage of CWR in dry matter. Lignin from the prepared CWR (5 mg ± 1 mg) was solubilized in 1 ml (V1) of acetyl bromide solution [acetyl bromide/acetic acid (1/4, v/v)] in a glass vial at 55°C for 2.5 h under shaking. Samples were then let to cool down at room temperature and 1.2 ml of NaOH 2M / acetic acid (9/50, v/v) was added in the vial. Then, 0.1 ml (V2) of this sample was transferred in 300 μl of 0.5 M hydroxylamine chlorhydrate and mixed with 1.4 ml of acetic acid. The absorbance (A280) of the samples was measured at 280 nm. Lignin content was calculated using the following formula: AcBL mg/g DW = 10 × (A280 × V1 × 3 × CWR%)/(20 × V2 × Masse sample in mg).

Glucose, fructose and sucrose were analyzed according to Gutjahr et al. (2013) with few adaptations. Twenty mg of the previous powder (<100 μm) were extracted with 1 ml 80% ethanol (v/v) for 30 min at 78°C, then centrifuged (10 min, 10000 rpm). The supernatant containing sugars was placed in 50 ml graduated flask. The pellet was re-suspended in 1 ml of 80% ethanol in same condition as previously, and the procedure was carried out three times. After homogenization and filtration with membrane filter at 0.22 μm, the mono and di saccharide contents were quantified using an HPAEC-PAD chromatography (Dionex, Salt Lake City, UT, United States). The separation was carried out by CarboPack PA1 column at 30°C with an isocratic elution of 150 mM sodium hydroxide.

### Data Analysis

Data were analyzed for two growth stages (end of stress and final harvest) using a linear mixed model:

V_ijkl_ = μ + Y_i_ + T_ij_ + G_k_ + α_ik_ + β_kj_ + a_il_ + b_ijl_ + e_ijkl,_ where: μ is the mean of the V variable,
Y_i_ is fixed effect associated with the i^th^ Year,
T_ij_ is fixed effect associated with the j^th^ Treatment in the i^th^ Year,
G_k_ is fixed effect associated with the k^th^ Genotype,
α_ik_ is the fixed effect associated with the interaction of the i^th^ Year and the k^th^ Genotype,
β_kj_ is the fixed effect associated with the interaction of the k^th^ Genotype and the j^th^ Treatment,
a_il_ is the random effect associated with the l^th^ block in the i^th^ Year,
b_ijl_ is the random effect associated with the j^th^ Treatment of the l^th^ block in the i^th^ Year,
e_ijkl_ is random error associated with the experimental unit in block l that received Treatment j for the Year i and the Genotype k.

The Treatment factor was nested in the Year factor as these factors could not be considered independent, as drought intensity depended of the year.

Comparison of means was performed using HSD-Tukey test. A critical value of α = 0.05 was used for the tests of significance. Principal Component Analysis (PCA) was used to analyze the correlations between the variables. PCA was performed on mean-centered data. Statistical analyses were performed using R (R Core Team, 2013). The PCA analyses were visualized using the XLSTAT software (Addinsoft, 2016).

### Internode Age Estimation

**FIGURE 2:**
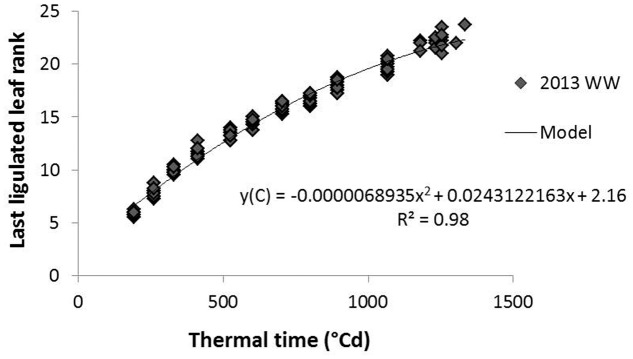
Relation between the number of ligulated leaves on the main stem and the cumulated thermal time from germination; example of 2013 average data on G1 and G4 hybrids in the well-watered (WW) treatment. The model fitted to the data is a polynomial equation here presented with corresponding r^2^.

Internode age was estimated in thermal time cumulated since their elongation onset. This was used to evaluate whether histochemical variables followed a regular pattern along internode aging whatever the internode rank. This estimation was based on the hypothesis that the growth of a given internode starts with the end of the expansion (ligulation) of the leaf of the same phytomer and ends with the beginning of the next internode growth (adapted from Nakamura et al., 2011). The measurement of the number of ligulated leaves along thermal time (averaged per replicate and per genotype in a given treatment) was used to estimate the thermal time at which a given internode rank started expanding (TT_init), by inverting the polynomial equation presented in **Figure 2**. The age of a given internode for a given genotype at a given sampling date was thereafter computed as the difference between the thermal time at sampling and TT_init.

## Results

### Impact of Water Deficit on Plant Morphology and Biomass Production

**FIGURE 3:**
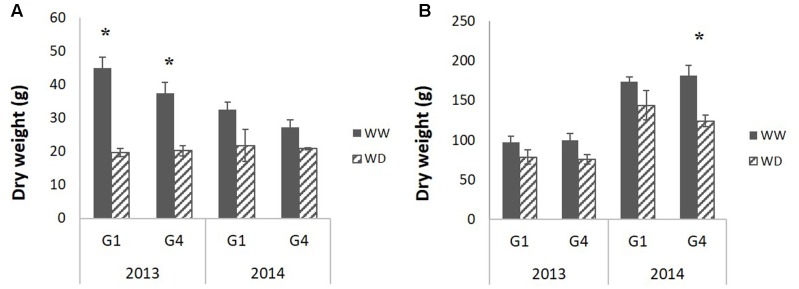
Average (of 12 plants per block, genotype and stage) stem dry weight measured **(A)** at the end of the water deficit period and **(B)** at final harvest for the two studied hybrids (G1: Biomass140, G4: RE1xAR4) on 2 years (2013, 2014) and two water conditions (well-watered: WW, 1-month water deficit during stem elongation: WD). Standard error bars are presented; black stars indicate significant treatment effects evaluated by ANOVA (*p*-value < 0.05).

The two hybrids showed similar phenology across years and treatments, which was a good prerequisite to provide a common developmental timeframe for the analyses (**Table 2**, 50% of plants flowering on September 7, ±5 days; Supplementary Table S1 provides corresponding coefficients of variation and standard errors). Most of the variables measured at plant level were statistically different between years (to the exception of PHT at the end of the stress period), plants being generally heavier in 2014 (**Table 2**). Shoot dry weight was negatively affected by drought at the end of the stress period (**Table 2**: reduction by 37 and 18% in 2013 and 2014 respectively) but it significantly recovered after re-watering and at final harvest, the plants in the WD treatment were only 13% (in 2013) and 15% (2014) lighter compared to the WW treatment. Stem dry weight, the main component of shoot dry weight, behaved similarly although it was more impacted at harvest (significant reduction of 16.5% in 2013 and 20.5% in 2014) (**Table 2** and **Figure 3**). Indeed, water deficit effect was significant both at the end of the stress period and at final harvest for stem dry weight and PHT as well. The stress effect on PHT was larger in 2013 than in 2014. Genotype effects were small although G4 was significantly taller and more impacted by water deficit compared to G1 (cf. G and GxT effects on PHT variable in **Table 2** and Supplementary Table S2 for average values per genotype in WW treatment). Leaf dry weight was significantly affected by water deficit at the end of the stress period (**Table 2**). It recovered after re-watering, as the drought effect was not significant at final harvest. The number of ligulated leaves was significantly reduced by drought (by two leaves) at the end of the stress but it fully recovered at the harvest stage after re-watering (**Table 2**). Accordingly, stem dry weight was reduced by drought but it largely recovered after re-watering which was associated with a total recovery of phytomer number, as represented by the number of ligulated leaves on the main stem at final harvest. The residual drought effect on final stem dry weight was thus the result of a reduction in internode size.

**Table 2:** Mean values and corresponding ANOVA results for morphological variables (24 plants per block and stage) measured at two stages (end of the water deficit period and final harvest), for two hybrids G (G1: Biomass140, G4: RE1xAR4), 2 years Y (2013, 2014) and two water treatments T (well-watered: WW and 1-month water deficit during stem elongation: WD).

		Mean								
							
		2013		2014		ANOVA				
				
Trait	Date	WW	WD	WW	WD	Y	G	T	GxY	GxT
**Dry weight (g)**										
Shoot	*End stress*	82.42	49.93	62.35	54.23	**0.008**	**0.045**	**0.000**	0.808	0.098
	*Harvest*	156.22	128.58	247.11	203.37	**0.000**	0.773	0.150	0.684	0.390
Stem	*End stress*	41.21	19.97	29.90	21.32	**0.003**	**0.044**	**0.000**	0.945	0.097
	*Harvest*	98.69	77.14	177.32	134.11	**0.000**	0.518	**0.041**	0.666	0.930
Leaf	*End stress*	41.21	29.96	32.45	32.91	**0.030**	0.051	**0.001**	0.581	0.108
	*Harvest*	50.30	44.02	53.75	54.35	0.260	0.323	0.198	0.811	0.913
**Plant Height Total (cm)**	*End stress*	227.53	116.92	233.47	154.39	0.660	**0.010**	**0.000**	**0.017**	**0.002**
	*Harvest*	306.38	219.53	283.28	232.65	**0.047**	**0.007**	**0.000**	0.776	**0.023**
**Last ligulated leaf rank**	*End stress*	16.8	14.3	18.7	16.9	**0.000**	0.277	**0.000**	**0.019**	0.329
	*Harvest*	21.9	22.6	23.7	24.2	0.220	0.580	**0.016**	0.509	0.381
**Internode Length (cm)**	*End stress*	30.19	26.86	27.92	12.79	0.371	0.526	**0.000**	0.948	0.526
	*Harvest*	23.69	18.56	21.04	20.00	0.325	0.216	**0.034**	0.998	**0.027**
**Internode Diameter (mm)**	*End stress*	16.70	15.90	18.76	18.14	**0.044**	0.766	0.651	0.642	0.913
	*Harvest*	13.79	13.03	17.40	18.03	**0.001**	0.504	0.802	0.181	0.985


*Internode length and diameter measured at rank 2 (end stress) and 4 (harvest) below the last ligulated leaf phytomer (p values < 0.05 are indicated in bold).*

### Impact of Water Deficit at the Internode Level

#### Internode Size

**FIGURE 4:**
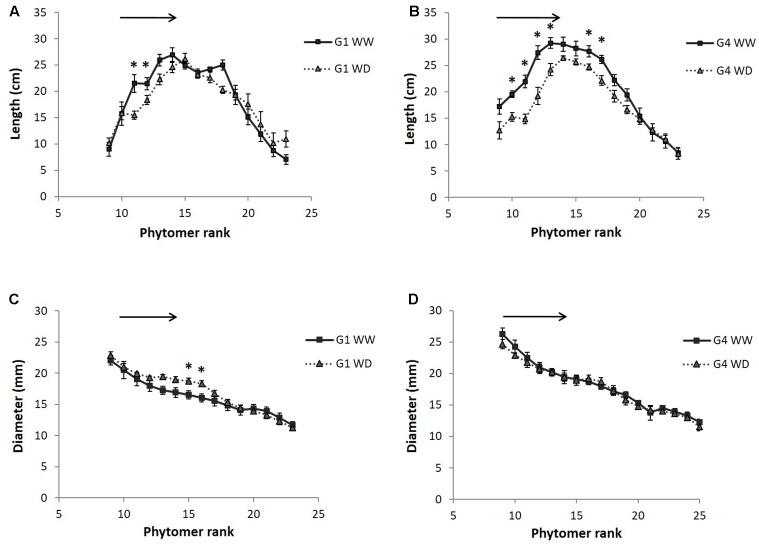
Internode length **(A,B)** and diameter **(C,D)** as a function of their rank along the main stem, measured in 2014 field experiment on two hybrids (G1: Biomass140, G4: RE1xAR4) and for two water conditions (WW: well-watered, WD: 1 month of water deficit during stem elongation). Each point on the curve is a mean of 12 internodes with standard error bars. The black arrows indicate internodes having grown during the water deficit period and the black stars internode ranks with significant treatment effects evaluated by ANOVA (*p*-value < 0.05).

The profile of internode length along the main stem is presented in **Figure 4** for 2014 (measurements in 2013 are compared to 2014 in **Table 2**). Internodes were significantly thicker but slightly shorter (only significant at the end of the stress period) in 2014 compared to 2013, at least for the internode ranks measured in 2013 (see Y effects in **Table 2**). For both genotypes, internode length increased from the first elongated internode (the 9th, from the bottom, i.e., belonging to phytomer 9) to the 16th internode, and then decreased progressively toward the top of the stem. Internode diameter decreased gradually from the bottom to the top of the stem. Internodes that expanded during the stress period had their length significantly reduced by water deficit (**Table 2** and **Figure 4**). By contrast, internode diameter was not impacted by water deficit for G4 (**Figure 4D**) and a non-significant positive effect was even observed for G1 internodes initiated during the stress period (ranks 13–16; **Figure 4C**). This effect of water deficit on internode diameter and the different response observed for the two hybrids was however, not confirmed by ANOVA (**Table 2**).

The reduction of stem dry weight and PHT at the end of the water deficit period (**Table 2** and **Figure 3**) was thus associated with a reduction of the length, but not the diameter, of internodes elongating during the water deficit period.

#### Dynamics of Internode Anatomy and Biochemical Composition

**Table 3** shows averages of histological and biochemical variables and related ANOVA results, for two internode positions that were observed in both years (Supplementary Table S3 provides corresponding coefficients of variation and standard errors). A strong year effect was observed at the end of the stress period as plants in 2013 had significantly higher values for traits related to lignin content (perSclZ1 and AcBL at *p* < 0.001; ADL at *p* < 0.05) and lower levels for traits related to cellulose (perbluZ2, *p* < 0.001) and soluble sugar (SS, *p* < 0.01) contents. No G effect was observed for histological variables, except on perZ1. However, G4 had higher lignin and cellulose (ADL, Cell_VS, Hemi_VS) and lower soluble sugar (SS) content than G1 at final harvest for the fourth internode situated below the last ligulated leaf (Supplementary Table S2).

**Table 3:** Mean values and corresponding ANOVA results for anatomical (perZ1: outer zone area (Z1) in % of internode section area, perSclZ1: percentage of sclerenchyma tissue (red stained) in % of Z1 area, densVBZ2: density of vascular bundles in central zone (Z2) in number of vascular bundles per mm^2^ and perBluZ2: percentage of blue tissue in % of Z2 area) and biochemical (ADL: Acid Detergent Lignin in %DW, Cell_VS: cellulose content in %DW, Hemi_VS: hemicellulose content in %DW, SS: soluble sugars in mg.g^-1^DW and AcBL: lignin content by acetyl bromide method in mg.g^-1^DW).

		Mean								
							
		2013		2014		ANOVA				
				
Trait	Date	WW	WD	WW	WD	Y	G	T	GxY	GxT
**perZ1** (% of section area)	*End stress*	16.93	18.22	15.48	11.21	0.621	**0.026**	0.085	**0.026**	0.138
	*Harvest*	18.40	18.32	17.40	18.03	0.276	**0.034**	0.871	**0.005**	0.341
**perSclZ1** (% of Z1 area)	*End stress*	49.98	39.85	21.03	18.82	**0.000**	0.176	0.213	**0.013**	0.998
	*Harvest*	61.42	57.91	50.29	42.99	0.672	0.700	0.484	**0.014**	0.289
**densVBZ2** (nb/mm^2^)	*End stress*	1.10	1.07	1.19	1.60	0.614	0.676	**0.033**	0.606	0.767
	*Harvest*	1.11	1.15	1.12	1.25	0.807	0.210	0.517	0.475	0.764
**perBluZ2** (% of Z2 area)	*End stress*	10.07	29.20	58.32	79.86	**0.000**	0.847	0.085	0.124	0.823
	*Harvest*	2.82	6.91	5.53	12.22	0.931	0.091	0.249	0.264	0.875
**ADL** (%DW)	*End stress*	4.84	3.81	3.89	1.79	**0.012**	0.651	**0.000**	0.964	0.599
	*Harvest*	5.54	5.71	5.11	4.67	0.365	**0.013**	0.882	0.372	0.447
**Cell_VS** (%DW)	*End stress*	39.47	34.62	39.21	27.60	0.386	0.899	**0.000**	0.550	0.554
	*Harvest*	36.17	37.52	35.92	33.94	0.624	**0.000**	0.581	0.234	0.828
**Hemi_VS** (%DW)	*End stress*	26.96	25.52	24.85	24.29	0.085	**0.026**	0.083	**0.000**	0.839
	*Harvest*	25.69	25.81	25.26	24.81	0.512	**0.001**	0.909	**0.029**	0.763
**SS** (mg.g^-1^ DW)	*End stress*	143.21	219.75	265.94	286.95	**0.003**	0.915	0.167	0.343	0.168
	*Harvest*	236.62	258.82	252.86	269.94	0.651	**0.008**	0.363	0.071	0.268
**AcBL** (mg.g^-1^ DW)	*End stress*	109.83	95.40	53.88	56.46	**0.000**	0.812	0.432	0.670	0.633
	*Harvest*	130.33	129.49	137.34	125.92	0.357	0.199	0.521	0.498	0.373


*Variables are measured at two stages (end of the water deficit period (*end stress*) and final harvest), at two internode levels on the main stem of two hybrids G (G1: Biomass140, G4: RE1xAR4), on 2 years in the field (2013, 2014) under two water treatments T (well-watered: WW, 1 month water deficit during stem elongation: WD). Each value in the table is the average of 24 internodes at rank 2 (end stress) and 4 (harvest) below the last ligulated leaf phytomer (*p*-values < 0.05 are indicated in bold).*

**FIGURE 5:**
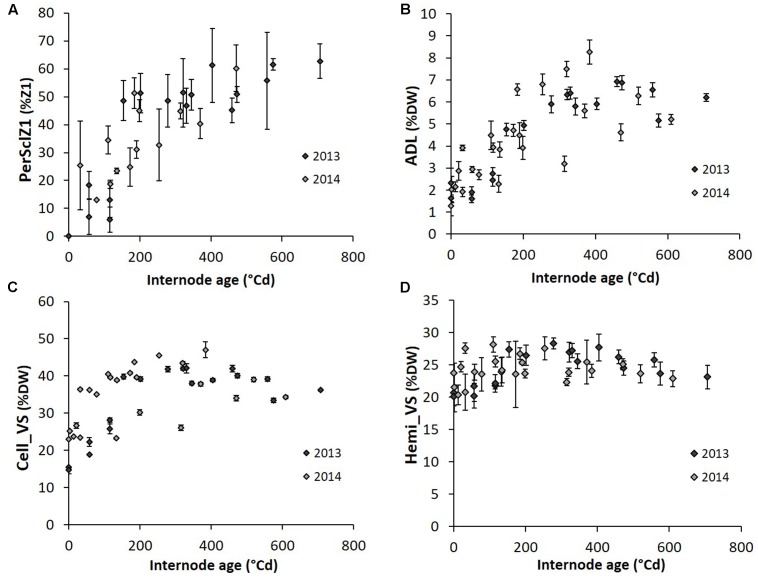
Dynamics of perSclZ1 **(A)**, ADL **(B)**, Cell_VS **(C)**, Hemi_VS **(D)** (perSclZ1: percentage of sclerenchyma tissue (red stained) in % of Z1 area, ADL: Acid Detergent Lignin in %DW, Cell_VS: cellulose content in %DW, Hemi_VS: hemicellulose content in %DW) as a function of sorghum internode age expressed in thermal time cumulated since their growth onset; average for 2 genotypes (G1: Biomass140 and G4: RE1xAR4) for each year (2013 and 2014), for the well-watered treatment only. Each point is the average (with standard error bars) of the three blocks. All internodes sampled at the end of the stress period and the final harvest were used (2013: 2nd and 4th ranks below the last ligulated leaf and 2014: last ligulated leaf rank and 2nd, 4th, and 6th ranks below).

G effects were observed only at the final harvest for biochemical traits (**Table 3** and Supplementary Table S1). By contrast, Y effects were detected only during plant growth. Also, the dynamic pattern of internode anatomical and biochemical variables along internode age in the well-watered treatment was analyzed for G1 and G4 together and separately for the years (**Figure 5**). PerSclZ1 increased gradually with internode age, reaching a plateau around 400°Cd (**Figure 5A**). In the central zone (Z2), the percentage of tissue stained in blue (perBluZ2) per unit area decreased gradually in an inversely proportional way compared to perSclZ1 (not shown). By contrast, the proportion of Z1 in the internode section area (perZ1) was fixed very early during internode development (not shown). The density (number per area unit) of vascular bundles (densVBZ2) decreased with internode age (likely resulting from the increase in diameter, not shown). The weight percentage of lignin (ADL method) and the lignin content (AcBL) followed a similar trend and increased with internode age and stabilized around 300°Cd (ADL: **Figure 5B** and AcBL: not shown), i.e., about 200–250°Cd after the end of internode elongation. By contrast, the weight percentage of hemicellulose (Hemi_VS) stabilized early during internode development (even before the end of internode elongation that happens after about 80°Cd; **Figure 5D**). Cell_VS followed a similar trend as lignin (ADL, AcBL) and reached a plateau around 300°Cd (**Figure 5B**).

Lignocellulose components therefore exhibited an increase along internode aging, attaining a plateau after the end of internode elongation. By contrast, the proportion of internode external vs. internal zones (perZ1) and internode hemicellulose content became constant soon after the onset of internode elongation.

#### Impact of Water Deficit on Internode Anatomical and Biochemical Traits

**FIGURE 6:**
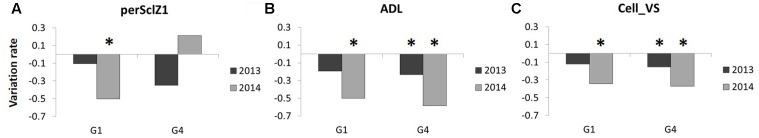
Variation of **(A)** perSclZ1, **(B)** ADL, **(C)** Cell_VS (perSclZ1: percentage of sclerenchyma tissue (red stained) in % of Z1 area, ADL: Acid Detergent Lignin in %DW, Cell_VS: cellulose content in %DW) between water deficit (WD) and well-watered (WW) treatments, measured at the end of the WD period on the internode of rank 2 below the last ligulated leaf phytomer; for 2 genotypes (G1: Biomass140 and G4: RE1xAR4) and 2 years (2013 and 2014); variation rate is computed as [(WD value) – (WW value)]/(WW value) using data averaged on the three blocks. ^∗^Indicates a significant water treatment effect (*p*-value < 0.05).

The effect of water deficit on internode anatomical and biochemical traits is presented in **Table 3**, and for three of the most plastic histochemical traits in **Figure 6**. The internodes analyzed for this purpose were, in 2013 and 2014: one having elongated during the stress period, i.e., at rank 2 below the last ligulated leaf phytomer at the end of the stress period (rank 13–16 from the bottom of the stem) and one having elongated after the water deficit period, i.e., at rank 4 below the flag leaf phytomer at final harvest (rank 17–21 from the bottom of the stem). ADL and Cell_VS were significantly reduced by water deficit at the end of the water deficit period (**Table 3**, **Figures 6A,B**, except not significant for G1 in 2013) and this reduction was generally stronger in 2014. Hemi_VS was not significantly reduced by water deficit (**Table 3**). Soluble sugar content (SS) was increased in internodes expanding during the water deficit and sampled at the end of the stress period, but this was not in all cases significant (**Table 3** and Supplementary Table S2). No significant water deficit effect was detected on perZ1, perSclZ1 and perBluZ2. DensVBZ2 was significantly increased by drought in internodes expanding during the water deficit period (**Table 3**). The internodes that expanded after the stress period were not affected by drought, except for a non-significant increase in perBluZ2 (**Table 3**).

Consequently, at the end of the water deficit period, ADL and Cell_VS were reduced and soluble sugars increased by water deficit in the internode expanding during this period, whereas Hemi_VS and histological variables were not changed. The internodes expanding after re-watering did not exhibit any residual effect of the water deficit period.

### Relationship between Biomass Component Traits

**FIGURE 7:**
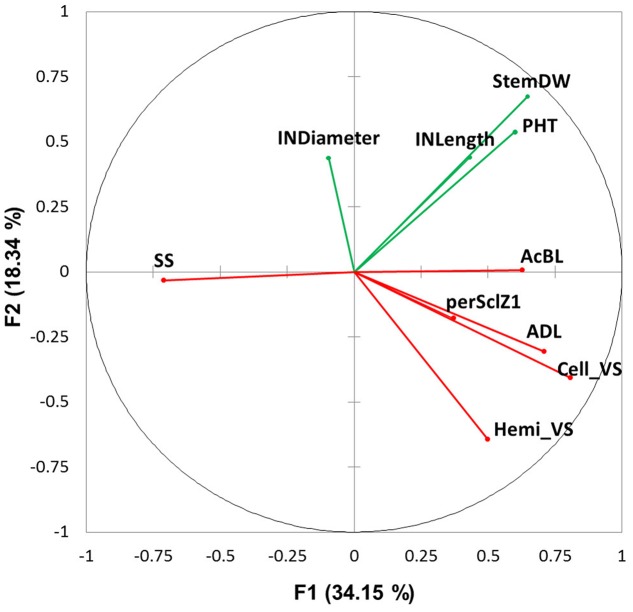
Principal component analysis using average variables measured in well-watered and water deficit treatments at plant (PHT: plant height; StemDW: stem dry weight) and internode level (INLength: internode length; INDiameter: internode diameter; ADL: Acid Detergent Lignin in %DW, Cell_VS: cellulose content in %DW, perSclZ1: percentage of sclerenchyma tissue (red stained) in % of Z1 area, SS: soluble sugars in mg.g^-1^DW and AcBL: lignin content by acetyl bromide method in mg.g^-1^DW) at final harvest (considering internode of rank 4 below the flag leaf). Morphological variables are in green and anatomical and biochemical variables in red. The axes F1 and F2 explained 52.50% of the total variation.

The covariation among the traits measured at different biological scales was analyzed with PCA, using data acquired at final harvest stage only, in the two water treatments and the 2 years (**Figure 7**). Most of the variability was captured by the opposition between SS (cos^2^ = 0.505) and fiber content (particularly AcBL, ADL and Cell_VS, with cos^2^ = 0.396, 0.502 and 0.652, respectively) on axis 1 (explaining 34% of the observed variation). Morphological variables mainly constituted axis 2 (StemDW on axis 2, cos^2^ = 0.453; internode length on axis 3, cos^2^ = 0.392).

The response to factors (genotype, treatment and year) of SS, fiber content (**Table 3** and **Figure 6**) and size (diameter, length, **Table 2**) of internodes was computed (**Table 4**). Internode length (but not diameter), perSclZ1 and ADL (AcBL to a lesser extent) were reduced by drought, whereas SS was increased. Interestingly, internode diameter but not length was greater in 2014 than in 2013. This was associated with slightly higher SS (5%) and reduced ADL and perSclZ1 (by -22% and -13%, respectively), but no change in AcBL. Between genotypes, G4 had smaller internode diameter (7% less than G1), less SS (-24%), more ADL and AcBL (+16 and +11%, respectively), but less perSclZ1 (-12%).

**Table 4:** Variation in internode anatomical and biochemical variables (INLength: internode length; INDiameter: internode diameter; perSclZ1: percentage of sclerenchyma tissue (red stained) in % of Z1 area, AcBL: lignin content by acetyl bromide method in mg⋅g^-1^DW, ADL: Acid Detergent Lignin in %DW and SS: soluble sugars in mg⋅g^-1^DW) in response to studied factors: genotype G (G4 value in reference to G1), treatment T (water deficit WD value in reference to well-watered WW), and year Y (2014 value in reference to 2013).

Response to	INLength	INDiameter	perSclZ1	AcBL	ADL	SS
T (WD vs. WW)	-0.30	-0.04	-0.20	-0.08	-0.34	0.27
Y (2014 vs. 2013)	-0.03	0.32	-0.22	0.02	-0.13	0.05
G (G4 vs. G1)	-0.01	-0.07	-0.12	0.16	0.11	-0.24


*The variation for a given variable and a given factor is computed as [(value) – (reference value)]/(reference value) using data average on the three blocks at the end of the stress period for T effect and at the final harvest for G and Y effects.*

Internode fiber content (ADL) and SS responded inversely to any given factor, whatever the factor effect on internode size. By contrast, the co-variation of traits related to lignin content depended on the factor: ADL and perSclZ1 varied similarly in response to T and Y (when internode size was affected); ADL and AcBL varied similarly in response to G effect, i.e., when no effect on internode size was observed.

## Discussion

### Drought Response and Recovery of Stem Biomass Depend on Internode Elongation

This study showed that the drought reduction of stem biomass was mainly related to reduced elongation and smaller final length of internodes that elongated during the drought. The full recovery of phytomer number after re-watering demonstrated that plant development *per se* was not affected by drought and did not contribute to shoot dry weight reduction (**Table 2** and **Figure 4**). Interestingly, the diameter of internodes elongating under water deficit was not significantly affected by drought and thus did not contribute to the reduction of stem biomass (**Table 2** and **Figure 4**). Ottman et al. (2001), Almodares et al. (2013), Hussein and Alva (2014), and Fracasso et al. (2016) attributed the reduction of stem biomass by drought to a reduction of plant height but did not relate this response to internode size and number. Although some reports showed that biomass production is more determined by stem height than diameter, particularly across genetic variation (George-Jaeggli et al., 2011; Salas Fernandez et al., 2015), the present study is not in line with some other studies: Almodares et al. (2013) reported for two genotypes that stem diameter was reduced proportionally to drought intensity and contributed to the reduction of stem biomass. In sugarcane, controversial results were reported regarding the effect of water deficit on stem diameter (Ramesh and Mahadevaswamy, 2000; Silva et al., 2008).

After re-watering and during the 15–20 days remaining before the end of stem elongation, stem biomass growth recovered remarkably for both genotypes and this was explained by the recovery of both the number and the size of internodes elongating during this period (**Table 2** and **Figure 4**). This was particularly the case for G1, whereas for G4, a depressive drought effect was observed on the length of the two to three first internodes elongating after rewatering (**Figure 4**, for 2014). Despite the recovery observed for both genotypes, stem biomass production remained significantly lower at final harvest in the water deficit treatment compared to the well-watered treatment. We suggest that not only stem elongation maintenance during drought but also recovery capacity are important traits for future phenotyping, genetic and breeding studies. The recovery process and its genotypic variability are poorly understood. Chenu et al. (2008) and Luquet et al. (2008) pointed out recovery for leaves in cereals. Our results have also implications for water management in biomass sorghum cropping systems (Habyarimana et al., 2004).

### Internode Morphological and Histochemical Traits Vary in Dynamics and Drought Sensitivity

#### All Internodes Follow a Common Pattern of Histochemical Construction

In this study it was shown that all internodes, regardless of position but for a given genotype and environment, followed the same pattern of biochemical and anatomical development, expressed along thermal time elapsing after onset of their elongation (**Figure 2**).

The elaboration of an internode developmental index relied on the estimation of internode elongation onset, based on the coordination demonstrated for cereals between leaf and internode elongation by Goto et al. (1994), Lafarge and Hammer (2002), and Nakamura et al. (2011). It is thus a proxy of internode age and enables to describe dynamics for internode histochemical development, which is original. This developmental index is a useful tool to define internode sampling dates in experiments and interpret observations.

#### Drought Sensitivity Differs among Histochemical Traits

The present study suggested that the drought sensitivity of internode biochemical and histological traits was related to their development (**Figure 4** vs. **Figure 5**). Traits related to lignification (ADL, AcBL, perSclZ1) as well as cellulose (Cell_VS), and soluble sugar contents (SS) presented a strong drought response, whereas those determined early along internode growth such as perZ1 and Hemi_VS did not show strong responses to drought. All these traits were not affected for internodes elongating after the drought period (**Table 3**). Unfortunately, the internode ranks sampled at the final harvest were not those that had elongated during the stress period and the recovery of the latter in terms of histochemical composition could not be analyzed. This will be interesting for a future study.

Soluble sugar content was increased by drought while cellulose and lignin contents (and related variables ADL, perSclZ1) were decreased along with internode length, in internodes expanding during the drought period and sampled at the end of this period. The increase of SS in sorghum stem under drought was previously reported for sweet sorghum and its extent was related to drought intensity and time of occurrence during crop cycle (Almodares et al., 2013; Tari et al., 2013; Ghate et al., 2017). The physiological role of an increase in SS in vegetative tissues under drought was already reported as serving osmoregulation and storage to ensure recovery after re-watering (Luquet et al., 2008; Rebolledo et al., 2013 in rice leaves; Robertson et al., 1999; Inman-Bamber and Smith, 2005 in sugarcane stem). This is not the case for lignin content reduction (Zegada-Lizarazu and Monti, 2013). The latter might rather be an emerging property of a reduction of C allocation to structural growth as represented by length reduction. Interestingly, the greater internode diameter in 2014 compared to 2013 (**Table 4**, +36%) was accompanied by an increase in SS and a reduction in lignin content (ADL, perSclZ1), similarly to the drought effect observed in this study, although 2014 had greater plant water supply (**Table 1**) which probably caused the greater biomass. There was no change in internode length and stem height between years. Greater biomass in 2014 can be explained by greater stem diameter and stem biomass density (estimated by dividing stem biomass by stem volume and equal to 0.13 and 0.17 g.cm^-3^ in 2013 and in 2014, respectively). Salas Fernandez et al. (2009) reported a positive correlation between stem biomass production and density across sorghum genotypes, but without exploring variation in histochemical composition. It will be interesting to further explore for a larger genetic diversity and contrasted water conditions if stem biomass density varies with histochemical variables and may serve as a proxy for them. This could be important in a phenotyping context with difficult access to high throughput laboratory analyses.

This study combined biochemical with histological characterization of internode biomass. Consistent relationships were found among these traits and their response to drought, but they were not as strong as expected. For example, lignin content measured either with the Van Soest method (ADL) or the acetyl bromide protocol (AcBL) was weakly correlated with the sclerenchyma proportion in the outer zone of the internode (perSclZ1). Several explanations can be suggested. First, histological traits captured information on an area basis which does not take into account variation in tissue density among the different zones within the internode. By contrast, biochemical variables were estimated on a dry weight basis and on bulk internode biomass. Second, the staining used for estimating tissue composition relies on competitive adsorption of the reagent by the tissue compounds and is neither highly specific nor quantitative. In addition, the quantification of the area characterized by a given color depends on the thresholds defined for image analysis and on the visual appraisal by the user (e.g., delimitation of Z1 and Z2 tissues). Lastly, the two histochemical traits related to lignin content did not respond similarly to the source of variation (G, Y, T), and ADL and perSclZ1 varied conjointly only when the size of the internode was also affected (length with T, diameter with Y effects). This result suggests that cell lignification in the outer part of the internode is related to internode structural growth and its plasticity in response to the environment.

### Implications for Biomass Sorghum Phenotyping and Crop Modeling

The present study suggested that internodes on the stem of a given genotype in a given environment follow the same histochemical development pattern (**Figure 5**). This means that the internodes at a given date along a given stem can be used to capture the developmental dynamics of internode biomass accumulation. This is valid, however, only if the plant was kept in a stable environment so that all internodes grew in similar conditions. This result is useful to simplify phenotyping and sampling protocols. The extrapolation of this approach to drought will be more difficult and require a stable and sufficiently long stress condition spanning the elongation of several consecutive internodes. This is being possible using controlled phenotyping facilities (e.g., Phenoarch^5^). Nevertheless, the throughput of biochemical and histological analyses will remain the limiting factor for phenotyping of large panels of genotypes (biochemical traits: Rebolledo et al., 2015; Turner et al., 2016; histological traits: Heckwolf et al., 2015) and dry-lab methodologies such as IR spectra (e.g., NIRS) are needed.

With respect to designing plant ideotypes combining optimal trait combinations for a given production and environment, crop modeling demonstrated its usefulness in the last decade (Martre et al., 2015; Rötter et al., 2015). For biomass sorghum and its targeted end-uses, implying both quantity and quality criteria, no crop model is today available to our knowledge. Recent adaptations and applications of APSIM crop model were carried out to evaluate morpho-physiological traits for sorghum biomass production (Hammer et al., 2010; George-Jaeggli et al., 2013) or maize growth and yield drought response (Reymond et al., 2004; Chenu et al., 2009). However, no modeling study took into account at the organ level the GxE involved in biomass sorghum production and its partitioning in non-structural vs. structural biomass (soluble sugar vs. fiber contents). This is now underway based on the present study (Fumey et al., 2016; Perrier et al., 2016), and will provide a crop growth model able to *in silico* explore trait combinations for biomass sorghum ideotypes for variable environments.

## Conclusion

This study analyzed morphological and histochemical traits underlying stem biomass accumulation in sorghum and its drought response at organ (internode) and tissue level. The maintenance and the recovery of internode elongation under and after water deficit were demonstrated to drive stem biomass production in drought prone environments. The histochemical traits evolving gradually along internode development were more sensitive to the environment than those fixed early after internode elongation onset. Internode soluble sugar and lignocellulose contents responded to drought in an opposite way. This covariation was also observed between genotypes and years. As these biochemical traits are essential to define biomass quality for end-uses, they should be considered in the biomass sorghum phenotyping and breeding context.

## Author Contributions

LP, DL, LR, AC-V, SJ, and DP wrote the manuscript and had primary responsibility for the final content. LP and DL integrated morpho-physiological, histological and biochemical data. LP, LR, and CD analyzed statistically the data. SR and DF collected and analyzed morpho-physiological data. AC-V, AS, DB, and DP collected and analyzed biochemical data. SJ and CB collected and analyzed histological data.

## Conflict of Interest Statement

The authors declare that the research was conducted in the absence of any commercial or financial relationships that could be construed as a potential conflict of interest.

## Footnotes

1 http://www.ragt.fr/

2 http://www.euralis-semences.fr

3 www6.montpellier.inra.fr/diascope/DIASCOPE/Diaphen

4 http://rsbweb.nih.gov/ij/download.html

5 https://www6.montpellier.inra.fr/lepse_eng/M3P/PHENOARCH-platform

## References

[B1] Addinsoft (2016). *XLSTAT V. 2016.04.32525: Data Analysis and Statistics Software for Microsoft Excel.* Paris: Addinsoft.

[B2] Almodares A. Hadi M. R. (2009). Production of bioethanol from sweet sorghum: a review. *Afr. J. Agric. Res.* 4 772–780.

[B3] Almodares A. Hotjatabady R. H. Mirniam E. (2013). Effects of drought stress on biomass and carbohydrate contents of two sweet sorghum cultivars. *J. Environ. Biol.* 34 585–589. 24617146

[B4] Anami S. E. Zhang L. M. Xia Y. Zhang Y. M. Liu Z. Q. Jing H. C. (2015). Sweet sorghum ideotypes: genetic improvement of the biofuel syndrome. *Food Energy Secur.* 4 159–177. 10.1002/fes3.63

[B5] Bennouna B. Lahrouni A. Bethenod O. Fournier C. Andrieu B. Khabba S. (2004). Development of maize internode under drought stress. *J. Agron. Crop Sci.* 3 94–102.

[B6] Berenguer M. J. Faci J. M. (2001). Sorghum (*Sorghum bicolor* L. Moench) yield compensation processes under different plant densities and variable water supply. *Eur. J. Agron.* 15 43–55. 10.1016/S1161-0301(01)00095-8

[B7] Borrell A. K. Mullet J. E. George-Jaeggli B. van Oosterom E. J. Hammer G. L. Klein P. E. (2014). Drought adaptation of stay-green sorghum is associated with canopy development, leaf anatomy, root growth, and water uptake. *J. Exp. Bot.* 65 6251–6263. 10.1093/jxb/eru232 25381433 PMC4223986

[B8] Chenu K. Chapman S. C. Hammer G. L. Mclean G. Salah H. B. H. Tardieu F. (2008). Short-term responses of leaf growth rate to water deficit scale up to whole-plant and crop levels: an integrated modelling approach in maize. *Plant Cell Environ.* 31 378–391. 10.1111/j.1365-3040.2007.01772.x 18088328

[B9] Chenu K. Chapman S. C. Tardieu F. McLean G. Welcker C. Hammer G. L. (2009). Simulating the yield impacts of organ-level quantitative trait loci associated with drought response in maize: a “gene-to-phenotype” modeling approach. *Genetics* 183 1507–1523. 10.1534/genetics.109.105429 19786622 PMC2787435

[B10] Crasta O. R. Xu W. W. Rosenow D. T. Mullet J. Nguyen H. T. (1999). Mapping of post-flowering drought resistance traits in grain sorghum: association between QTLs influencing premature senescence and maturity. *Mol. Gen. Genet. MGG* 262 579–588. 10.1007/s004380051120 10589847

[B11] De Alencar Figueiredo L. F. Sine B. Chantereau J. Mestres C. Fliedel G. Rami J. F. (2010). Variability of grain quality in sorghum: association with polymorphism in Sh2, Bt2, SssI, Ae1, Wx and O2. *Theor. Appl. Genet.* 121 1171–1185. 10.1007/s00122-010-1380-z 20567801

[B12] Delalande M. Regnard J. L. Costes E. Reymond M. Luquet D. Fabre D. (2015). *DiaPHEN: Field Platform to Analyze Mechanisms of Drought Tolerance at Field Level.* Montpellier: EUCARPIA.

[B13] Dugas D. V. Monaco M. K. Olson A. Klein R. R. Kumari S. Ware D. (2011). Functional annotation of the transcriptome of *Sorghum bicolor* in response to osmotic stress and abscisic acid. *BMC Genomics* 12:514 10.1186/1471-2164-12-514 PMC3219791 22008187

[B14] Ferreira T. A. Rasband W. (2010). *The ImageJ User Guide—Version 1.43.* Available at: http://rsbweb.nih.gov/ij/docs/user-guide.pdf.

[B15] Fracasso A. Trindade L. Amaducci S. (2016). Drought tolerance strategies highlighted by two *Sorghum bicolor* races in a dry-down experiment. *J. Plant Physiol.* 190 1–14. 10.1016/j.jplph.2015.10.009 26624226

[B16] Fu T. Ko J. Wall G. W. Pinter P. J. Kimball B. A. Ottman M. J. (2016). Simulation of climate change impacts on grain sorghum production grown under free air CO2 enrichment. *Int. Agrophys.* 30 311–322. 10.1515/intag-2016-0007

[B17] Fukushima R. S. Hatfield R. D. (2001). Extraction and isolation of lignin for utilization as a standard to determine lignin concentration using the acetyl bromide spectrophotometric method. *J. Agric. Food Chem.* 49 3133–3139. 10.1021/jf010449r 11453742

[B18] Fukushima R. S. Kerley M. S. Ramos M. H. Porter J. H. Kallenbach R. L. (2015). Comparison of acetyl bromide lignin with acid detergent lignin and Klason lignin and correlation with in vitro forage degradability. *Anim. Feed Sci. Technol.* 201 25–37. 10.1016/j.anifeedsci.2014.12.007

[B19] Fumey D. Soulié J.-C. Fabre D. Luquet D. (2016). “Exploring modeling concepts to deal with carbon source-sink relationships in EcoMeristem: implications for analyzing the phenotypic variability of biomass-sorghum,” in *Communication at FSPMA2016 International Conference on Functional-Structural Plant Growth Modeling, Simulation, Visualization and Applications, IEEE*, Qingdao.

[B20] Garofalo P. Rinaldi M. (2011). Water-use efficiency of irrigated biomass sorghum in a Mediterranean environment. *Span. J. Agric. Res.* 11 1153–1169. 10.5424/sjar/2013114-4147

[B21] George-Jaeggli B. Jordan D. R. Van Oosterom E. J. Broad I. J. Hammer G. L. (2013). Sorghum dwarfing genes can affect radiation capture and radiation use efficiency. *Field Crops Res.* 149 283–290. 10.1016/j.fcr.2013.05.005

[B22] George-Jaeggli B. Jordan D. R. Van Oosterom E. J. Hammer G. L. (2011). Decrease in sorghum grain yield due to the dw3 dwarfing gene is caused by reduction in shoot biomass. *Field Crops Res.* 124 231–239. 10.1016/j.fcr.2011.07.005

[B23] Ghate T. Deshpande S. Bhargava S. (2017). Accumulation of stem sugar and its remobilisation in response to drought stress in a sweet sorghum genotype and its near-isogenic lines carrying different stay-green loci. *Plant Biol.* 19 396–405. 10.1111/plb.12538 28032438

[B24] Goto Y. Nakamura S. Sakai K. Hoshikawa K. (1994). Analysis of elongation and thickening of internodes in sweet sorghum (*Sorghum bicolor* Moench). *Jpn. J. Crop Sci.* 63 473–479. 10.1626/jcs.63.473

[B25] Gutjahr S. Clément-Vidal A. Soutiras A. Sonderegger N. Braconnier S. Dingkuhn M. (2013). Grain, sugar and biomass accumulation in photoperiod-sensitive sorghums. II. Biochemical processes at internode level and interaction with phenology. *Funct. Plant Biol.* 40 355–368. 10.1071/FP12177 32481113

[B26] Habyarimana E. Laureti D. De Ninno M. Lorenzoni C. (2004). Performances of biomass sorghum [*Sorghum bicolor* (L.) Moench] under different water regimes in Mediterranean region. *Ind. Crops Prod.* 20 23–28. 10.1016/j.indcrop.2003.12.019

[B27] Hammer G. L. van Oosterom E. McLean G. Chapman S. C. Broad I. Harland P. (2010). Adapting APSIM to model the physiology and genetics of complex adaptive traits in field crops. *J. Exp. Bot.* 61 2185–2202. 10.1093/jxb/erq095 20400531

[B28] Heckwolf S. Heckwolf M. Kaeppler S. M. de Leon N. Spalding E. P. (2015). Image analysis of anatomical traits in stalk transections of maize and other grasses. *Plant Methods* 11 26 10.1186/s13007-015-0070-x PMC4404653 25901177

[B29] Hussein M. M. Alva A. K. (2014). Growth, yield and water use efficiency of forage sorghum as affected by NPK fertilizer and deficit irrigation. *Am. J. Plant Sci.* 5 2134–2140. 10.4236/ajps.2014.513225

[B30] Inman-Bamber N. G. Smith D. M. (2005). Water relations in sugarcane and response to water deficits. *Field Crops Res.* 92 185–202.

[B31] Jaffuel S. Vom Brocke K. Hamadou T. V. Dardou A. Vidal A. Trouche G. (2016). Anatomical features of an African sorghum landrace adapted to flooded conditions. *Aust. J. Crop Sci.* 10 1489–1495. 10.21475/ajcs.2016.10.10.p7867

[B32] Jung H. G. Casler M. D. (2006). Maize stem tissues: impact of development on cell wall degradability. *Crop Sci.* 46 1801–1809. 10.2135/cropsci2006.02-0086

[B33] Jung H. J. G. (2003). Maize stem tissues: ferulate deposition in developing internode cell walls. *Phytochemistry* 63 543–549. 10.1016/S0031-9422(03)00221-8 12809714

[B34] Kim H. K. Luquet D. Van Oosterom E. Dingkuhn M. Hammer G. (2010). Regulation of tillering in sorghum: genotypic effects. *Ann. Bot.* 106 69–78. 10.1093/aob/mcq080 20430784 PMC2889794

[B35] Lafarge T. A. Hammer G. L. (2002). Predicting plant leaf area production: shoot assimilate accumulation and partitioning, and leaf area ratio, are stable for a wide range of sorghum population densities. *Field Crops Res.* 77 137–151. 10.1016/S0378-4290(02)00085-0

[B36] Luquet D. Clément-Vidal A. Fabre D. This D. Sonderegger N. Dingkuhn M. (2008). Orchestration of transpiration, growth and carbohydrate dynamics in rice during a dry-down cycle. *Funct. Plant Biol.* 35 689–704. 10.1071/FP08027 32688823

[B37] Mace E. S. Tai S. Gilding E. K. Li Y. Prentis P. J. Bian L. (2013). Whole-genome sequencing reveals untapped genetic potential in Africa’s indigenous cereal crop sorghum. *Nat. Commun.* 4 2320 10.1038/ncomms3320 PMC3759062 23982223

[B38] Martre P. Wallach D. Asseng S. Ewert F. Jones J. W. Rötter R. P. (2015). Multimodel ensembles of wheat growth: many models are better than one. *Glob. Change Biol.* 21 911–925. 10.1111/gcb.12768 25330243

[B39] Matos D. A. Whitney I. P. Harrington M. J. Hazen S. P. (2013). Cell walls and the developmental anatomy of the *Brachypodium distachyon* stem internode. *PLoS ONE* 8:e80640 10.1371/journal.pone.0080640 PMC3836760 24278300

[B40] Murray S. C. Rooney W. L. Hamblin M. T. Mitchell S. E. Kresovich S. (2009). Sweet sorghum genetic diversity and association mapping for brix and height. *Plant Genome* 2 48–62. 10.1007/s00122-009-1155-6

[B41] Murray S. C. Rooney W. L. Mitchell S. E. Sharma A. Klein P. E. Mullet J. E. (2008). Genetic improvement of sorghum as a biofuel feedstock: II. QTL for stem and leaf structural carbohydrates. *Crop Sci.* 48 2180–2193. 10.2135/cropsci2008.01.0068

[B42] Nakamura S. Nakajima N. Nitta Y. Goto Y. (2011). Analysis of successive internode growth in sweet sorghum using leaf number as a plant age indicator. *Plant Prod. Sci.* 14 299–306. 10.1626/pps.14.299

[B43] Ottman M. J. Kimball B. A. Pinter P. J. Wall G. W. Vanderlip R. L. Leavitt S. W. (2001). Elevated CO2 increases sorghum biomass under drought conditions. *New Phytol.* 150 261–273. 10.1016/j.jplph.2011.05.005

[B44] Perrier L. Rouan L. Fumey D. Soulié J.-C. Luquet D. (2016). “Analysing EcoMeristem model capacity to capture phenotypic variability of biomass-sorghum: validation, sensitivity analysis and ideotype exploration,” in *Poster at the Session Presented at FSPMA2016 International Conference on Functional-Structural Plant Growth Modeling, Simulation, Visualization and Applications, IEEE*, Qingdao.

[B45] Potgieter A. B. Lobell D. B. Hammer G. L. Jordan D. R. Davis P. Brider J. (2016). Yield trends under varying environmental conditions for sorghum and wheat across Australia. *Agric. For. Meteorol.* 228 276–285. 10.1016/j.agrformet.2016.07.004

[B46] R Core Team (2013) *R: A Language and Environment for Statistical Computing*. Vienna: R Foundation for Statistical computing.

[B47] Ramesh P. Mahadevaswamy M. (2000). Effect of formative phase drought on different classes of shoots, shoot mortality, cane attributes, yield and quality of four sugarcane cultivars. *J. Agron. Crop Sci.* 185 249–258. 10.1046/j.1439-037x.2000.00399.x

[B48] Rebolledo M. C. Dingkuhn M. Courtois B. Gibon Y. Clément-Vidal A. Cruz D. F. (2015). Phenotypic and genetic dissection of component traits for early vigour in rice using plant growth modelling, sugar content analyses and association mapping. *J. Exp. Bot.* 66 5555–5566. 10.1093/jxb/erv258 26022255 PMC4585419

[B49] Rebolledo M. C. Luquet D. Courtois B. Henry A. Soulié J. C. Rouan L. (2013). Can early vigour occur in combination with drought tolerance and efficient water use in rice genotypes? *Funct. Plant Biol.* 40 582–594. 10.1071/FP12312 32481132

[B50] Reymond M. Muller B. Tardieu F. (2004). Dealing with the genotype × environment interaction via a modelling approach: a comparison of QTLs of maize leaf length or width with QTLs of model parameters. *J. Exp. Bot.* 55 2461–2472. 10.1093/jxb/erh200 15286140

[B51] Richie W. R. McBee G. G. (1991). Structural components in sorghum stem biomass. *Bioresour. Technol.* 38 15–22. 10.3389/fpls.2016.00822

[B52] Robertson M. J. Inman-Bamber N. G. Muchow R. C. Wood A. W. (1999). Physiology and productivity of sugarcane with early and mid-season water deficit. *Field Crops Res.* 64 211–227. 10.1016/S0378-4290(99)00042-8

[B53] Rooney W. D. Johnson G. Li X. Cohen E. R. Kim S. G. Ugurbil K. (2007). Magnetic field and tissue dependencies of human brain longitudinal 1H2O relaxation in vivo. *Magn. Reson. Med.* 57 308–318. 10.1002/mrm.21122 17260370

[B54] Rötter R. P. Tao F. Höhn J. G. Palosuo T. (2015). Use of crop simulation modelling to aid ideotype design of future cereal cultivars. *J. Exp. Bot.* 66 3463–3476. 10.1093/jxb/erv098 25795739

[B55] Salas Fernandez M. G. Becraft P. W. Yin Y. Lübberstedt T. (2009). From dwarves to giants? Plant height manipulation for biomass yield. *Trends Plant Sci.* 14 454–461. 10.1016/j.tplants.2009.06.005 19616467

[B56] Salas Fernandez M. G. S. Strand K. Hamblin M. T. Westgate M. Heaton E. Kresovich S. (2015). Genetic analysis and phenotypic characterization of leaf photosynthetic capacity in a sorghum (Sorghum spp.) diversity panel. *Genet. Resour. Crop Evol.* 62 939–950. 10.1007/s10722-014-0202-6

[B57] Silva M. D. A. Silva J. A. G. D. Enciso J. Sharma V. Jifon J. (2008). Yield components as indicators of drought tolerance of sugarcane. *Sci. Agric.* 65 620–627. 10.1590/S0103-90162008000600008

[B58] Srivastava A. Kumar S. N. Aggarwal P. K. (2010). Assessment on vulnerability of sorghum to climate change in India. *Agric Ecosyst. Environ.* 138 160–169. 10.1016/j.agee.2010.04.012

[B59] Tari I. Laskay G. Takács Z. Poór P. (2013). Response of sorghum to abiotic stresses: a review. *J. Agron. Crop Sci.* 199 264–274. 10.1111/jac.12017

[B60] Tolivia D. Tolivia J. (1987). Fasga: a new polychromatic method for simultaneous and differential staining of plant tissues. *J. Microsc.* 148 113–117. 10.1111/j.1365-2818.1987.tb02859.x

[B61] Tovignan T. K. Fonceka D. Ndoye I. Cisse N. Luquet D. (2016). The sowing date and post-flowering water status affect the sugar and grain production of photoperiodic, sweet sorghum through the regulation of sink size and leaf area dynamics. *Field Crops Res.* 192 67–77. 10.1016/j.fcr.2016.04.015

[B62] Trouche G. Bastianelli D. Hamadou T. C. Chantereau J. Rami J. F. Pot D. (2014). Exploring the variability of a photoperiod-insensitive sorghum genetic panel for stem composition and related traits in temperate environments. *Field Crops Res.* 166 72–81. 10.1016/j.fcr.2014.06.008

[B63] Tsuchihashi N. Goto Y. (2005). Internode characteristics of sweet Sorghum (*Sorghum bicolor* (L.) Moench) during Dry and Rainy Seasons in Indonesia. *Plant Prod. Sci.* 8 601–607. 10.1626/pps.8.601

[B64] Tuinstra M. R. Grote E. M. Goldsbrough P. B. Ejeta G. (1997). Genetic analysis of post-flowering drought tolerance and components of grain development in *Sorghum bicolor* (L.) Moench. *Mol. Breed.* 3 439–448. 10.1023/A:1009673126345

[B65] Turner M. F. Heuberger A. L. Kirkwood J. S. Collins C. C. Wolfrum E. J. Broeckling C. D. (2016). Non-targeted metabolomics in diverse sorghum breeding lines indicates primary and secondary metabolite profiles are associated with plant biomass accumulation and photosynthesis. *Front. Plant Sci.* 7:953 10.3389/fpls.2016.00953 PMC4939745 27462319

[B66] Vadez V. Deshpande S. P. Kholova J. Hammer G. L. Borrell A. K. Talwar H. S. (2011). Stay-green quantitative trait loci’s effects on water extraction, transpiration efficiency and seed yield depend on recipient parent background. *Funct. Plant Biol.* 38 553–566. 10.1071/FP11073 32480908

[B67] Van Soest P. V. Robertson J. B. Lewis B. A. (1991). Methods for dietary fiber, neutral detergent fiber, and nonstarch polysaccharides in relation to animal nutrition. *J. Dairy Sci.* 74 3583–3597. 10.3168/jds.S0022-0302(91)78551-2 1660498

[B68] Vasilakoglou I. Dhima K. Karagiannidis N. Gatsis T. (2011). Sweet sorghum productivity for biofuels under increased soil salinity and reduced irrigation. *Field Crops Res.* 120 38–46. 10.1016/j.fcr.2010.08.011

[B69] Zegada-Lizarazu W. Monti A. (2012). Water uptake efficiency and above-and belowground biomass development of sweet sorghum and maize under different water regimes. *Plant Soil* 351 47–60. 10.1007/s11104-011-0928-2

[B70] Zegada-Lizarazu W. Monti A. (2013). Photosynthetic response of sweet sorghum to drought and re-watering at different growth stages. *Physiol. Plant.* 149 56–66. 10.1111/ppl.12016 23198740

