# Supplementary material for: Plasticity of Sorghum Stem Biomass Accumulation in Response to Water Deficit: A Multiscale Analysis from Internode Tissue to Plant Level
Source: Front Plant Sci. 2017 Sep 1;8:1516. doi: 10.3389/fpls.2017.01516 (PMC5585773; doi:10.3389/fpls.2017.01516)
Supplement: Supplementary file 1 [file Table_1.DOCX]

**Supplementary Table S1:** Mean values and corresponding coefficients of variation (CV) and standard errors (SE) for morphological variables (24 plants per block and stage) measured at 2 stages (end of the water deficit period and final harvest), for two hybrids G (G1: Biomass140, G4: RE1xAR4), 2 years Y (2013, 2014) and 2 water treatments T (well-watered: WW and one-month water deficit during stem elongation: WD). Internode length and diameter measured at rank 2 (end of stress) and 4 (final harvest) below the last ligulated leaf phytomer.

| **Trait** | **Date** | | **2013** | | | | | | **2014** | | | | | |
| --- | --- | --- | --- | --- | --- | --- | --- | --- | --- | --- | --- | --- | --- | --- |
|  |  |  | **Mean** | | **CV** | | **SE** | | **Mean** | | **CV** | | **SE** | |
|  |  |  | **WW** | **WD** | **WW** | **WD** | **WW** | **WD** | **WW** | **WD** | **WW** | **WD** | **WW** | **WD** |
| **Dry weight (g)** | |  |  |  |  |  |  |  |  |  |  |  |  |  |
| Shoot | *End stress* | | **82.42** | **49.93** | 0.176 | 0.128 | 5.113 | 2.265 | **62.35** | **54.23** | 0.151 | 0.169 | 3.831 | 0.735 |
|  | *Harvest* | | **156.22** | **128.58** | 0.145 | 0.162 | 7.995 | 7.343 | **247.11** | **203.37** | 0.093 | 0.157 | 9.392 | 13.04 |
| Stem | *End stress* | | **41.21** | **19.97** | 0.176 | 0.128 | 2.556 | 0.906 | **29.90** | **21.32** | 0.150 | 0.250 | 1.833 | 2.177 |
|  | *Harvest* | | **98.69** | **77.14** | 0.156 | 0.188 | 5.432 | 5.136 | **177.32** | **134.11** | 0.094 | 0.183 | 6.829 | 10.03 |
| Leaf | *End stress* | | **41.21** | **29.96** | 0.176 | 0.128 | 2.556 | 1.359 | **32.45** | **32.91** | 0.155 | 0.127 | 2.048 | 1.704 |
|  | *Harvest* | | **50.30** | **44.02** | 0.112 | 0.116 | 1.983 | 1.798 | **53.75** | **54.35** | 0.122 | 0.104 | 2.684 | 2.303 |
| **Plant Height Total (cm)** | *End stress* | | **227.53** | **116.92** | 0.074 | 0.117 | 2.976 | 2.418 | **233.47** | **154.39** | 0.135 | 0.093 | 7.409 | 3.376 |
|  | *Harvest* | | **306.38** | **219.53** | 0.115 | 0.131 | 6.205 | 5.098 | **283.28** | **232.65** | 0.094 | 0.068 | 6.632 | 3.811 |
| **Last ligulated leaf rank** | *End stress* | | **16.8** | **14.3** | 0.050 | 0.080 | 0.149 | 0.202 | **18.7** | **16.9** | 0.064 | 0.074 | 0.280 | 0.297 |
|  | *Harvest* | | **21.9** | **22.6** | 0.067 | 0.091 | 0.232 | 0.287 | **23.7** | **24.2** | 0.076 | 0.045 | 0.395 | 0.219 |
| **Internode Length (cm)** | *End stress* | | **30.19** | **26.86** | 0.111 | 0.118 | 1.188 | 1.199 | **27.92** | **12.79** | 0.128 | 0.256 | 1.463 | 1.336 |
|  | *Harvest* | | **23.69** | **18.56** | 0.204 | 0.223 | 1.706 | 1.465 | **21.04** | **20.00** | 0.124 | 0.264 | 1.069 | 2.158 |
| **Internode Diameter (mm)** | *End stress* | | **16.70** | **15.90** | 0.131 | 0.061 | 0.772 | 0.369 | **18.76** | **18.14** | 0.044 | 0.080 | 0.334 | 0.593 |
|  | *Harvest* | | **13.79** | **13.03** | 0.142 | 0.061 | 0.694 | 0.283 | **17.40** | **18.03** | 0.103 | 0.158 | 0.734 | 1.163 |
