# Supplementary material for: Plasticity of Sorghum Stem Biomass Accumulation in Response to Water Deficit: A Multiscale Analysis from Internode Tissue to Plant Level
Source: Front Plant Sci. 2017 Sep 1;8:1516. doi: 10.3389/fpls.2017.01516 (PMC5585773; doi:10.3389/fpls.2017.01516)
Supplement: Supplementary file 2 [file Table_2.DOCX]

**Supplementary Table S2:** Mean values for morphological, anatomical and biochemical variables (24 plants per block and stage) measured at final harvest, for two hybrids G (G1: Biomass140, G4: RE1xAR4), 2 years Y (2013, 2014) in the well-watered treatment only. Internode length, diameter, anatomical and biochemical variables correspond to measurements on the internode of rank 4 below the last ligulated leaf phytomer. (perZ1: outer zone area (Z1) in % of internode section area, perSclZ1: percentage of sclerenchyma tissue (red stained) in % of Z1 area, densVBZ2: density of vascular bundles in central zone (Z2) in number of vascular bundles per mm² and perBluZ2: percentage of blue tissue in % of Z2 area) and biochemical (ADL: Acid Detergent Lignin in %DW, Cell_VS: cellulose content in %DW, Hemi_VS: hemicellulose content in %DW, SS: soluble sugars in mg.g^-1^DW and AcBL: lignin content by acetyl bromide method in mg.g^-1^DW).

| **Trait** | **Mean** | | | |
| --- | --- | --- | --- | --- |
|  | **2013** | | **2014** | |
|  | **G1** | **G4** | **G1** | **G4** |
| **Dry weight (g)** |  |  |  |  |
| Shoot | 155.64 | 156.79 | 246.45 | 247.76 |
| Stem | 97.21 | 100.17 | 173.55 | 181.15 |
| Leaf | 51.22 | 49.38 | 56.84 | 50.65 |
| **Plant Height Total (cm)** | 294.81 | 317.94 | 270.94 | 295.63 |
| **Last ligulated leaf rank** | 20.5 | 23.7 | 23 | 24.3 |
| **Internode Length (cm)** | 26.25 | 21.13 | 21.25 | 20.83 |
| **Internode Diameter (mm)** | 14.07 | 13.52 | 18.75 | 16.05 |
| **perZ1** (% of section area) | 16.90 | 19.90 | 18.75 | 16.05 |
| **perSclZ1** (% of Z1 area) | 61.58 | 61.26 | 60.21 | 40.37 |
| **densVBZ2**  (nb.mm^-^²) | 1.17 | 1.04 | 1.12 | 1.12 |
| **perBluZ2** (% of Z2 area) | 4.24 | 1.4 | 8.17 | 2.89 |
| **ADL** (% DW) | 5.16 | 5.91 | 4.62 | 5.59 |
| **Cell_VS** (% DW) | 30.91 | 36.16 | 32.40 | 35.31 |
| **Hemi_VS** (% DW) | 23.68 | 27.70 | 25.09 | 25.44 |
| **SS** (mg.g^-1^ DW) | 267.45 | 205.78 | 271.11 | 234.60 |
| **AcBL** (mg. g^-1^ DW) | 125.62 | 135.04 | 129.12 | 145.56 |
