# Supplementary material for: Plasticity of Sorghum Stem Biomass Accumulation in Response to Water Deficit: A Multiscale Analysis from Internode Tissue to Plant Level
Source: Front Plant Sci. 2017 Sep 1;8:1516. doi: 10.3389/fpls.2017.01516 (PMC5585773; doi:10.3389/fpls.2017.01516)
Supplement: Supplementary file 3 [file Table_3.DOCX]

**Supplementary Table S3:** Mean values and corresponding coefficients of variation (CV) and standard errors (SE) for anatomical (perZ1: outer zone area (Z1) in % of internode section area, perSclZ1: percentage of sclerenchyma tissue (red stained) in % of Z1 area, densVBZ2: density of vascular bundles in central zone (Z2) in number of vascular bundles per mm² and perBluZ2: percentage of blue tissue in % of Z2 area) and biochemical (ADL: Acid Detergent Lignin in %DW, Cell_VS: cellulose content in %DW, Hemi_VS: hemicellulose content in %DW, SS: soluble sugars in mg.g^-1^DW and AcBL: lignin content by acetyl bromide method in mg.g^-1^DW). Variables measured at 2 stages (end of the water deficit period *end stress*, and final harvest) on two hybrids (G1: Biomass140, G4: RE1xAR4), on 2 years in the field (2013, 2014) under 2 water treatments (well-watered: WW, 1 month water deficit during stem elongation: WD). Each value in the table is the average of 24 internodes at rank 2 (*end stress*) and 4 (*harvest*) below the last ligulated leaf phytomer.

| **Trait** | **Date** | **2013** | | | | | | **2014** | | | | | | |
| --- | --- | --- | --- | --- | --- | --- | --- | --- | --- | --- | --- | --- | --- | --- |
|  |  | **Mean** | | **CV** | | **SE** | | **Mean** | | **CV** | | | **SE** | |
|  |  | **WW** | **WD** | **WW** | **WD** | **WW** | **WD** | **WW** | **WD** | | **WW** | **WD** | **WW** | **WD** |
| **perZ1** (% of section area) | *End stress* | **16.93** | **18.22** | 0.257 | 0.091 | 1.536 | 0.626 | **15.48** | **11.21** | | 0.158 | 0.354 | 0.996 | 1.621 |
|  | *Harvest* | **18.40** | **18.32** | 0.109 | 0.072 | 0.707 | 0.468 | **17.40** | **18.03** | | 0.103 | 0.158 | 0.734 | 1.163 |
| **perSclZ1** (% of Z1 area) | *End stress* | **49.98** | **39.85** | 0.255 | 0.218 | 4.501 | 3.288 | **21.03** | **18.82** | | 0.150 | 0.607 | 1.288 | 4.666 |
|  | *Harvest* | **61.42** | **57.91** | 0.126 | 0.141 | 2.739 | 2.879 | **50.29** | **42.99** | | 0.254 | 0.409 | 5.205 | 7.186 |
| **densVBZ2** (nb/mm²) | *End stress* | **1.10** | **1.07** | 0.219 | 0.102 | 0.085 | 0.041 | **1.19** | **1.60** | | 0.130 | 0.115 | 0.063 | 0.075 |
|  | *Harvest* | **1.11** | **1.15** | 0.159 | 0.085 | 0.062 | 0.034 | **1.12** | **1.25** | | 0.088 | 0.111 | 0.040 | 0.057 |
| **perBluZ2** (% of Z2 area) | *End stress* | **10.07** | **29.20** | 0.743 | 0.878 | 2.646 | 9.694 | **58.32** | **79.86** | | 0.280 | 0.136 | 6.658 | 4.443 |
|  | *Harvest* | **2.82** | **6.91** | 1.047 | 1.130 | 1.044 | 2.761 | **5.53** | **12.22** | | 1.077 | 0.315 | 2.431 | 1.571 |
| **ADL** (%DW) | *End stress* | **4.84** | **3.81** | 0.078 | 0.189 | 0.047 | 0.090 | **3.89** | **1.79** | | 0.062 | 0.258 | 0.040 | 0.077 |
|  | *Harvest* | **5.54** | **5.71** | 0.100 | 0.112 | 0.069 | 0.080 | **5.11** | **4.67** | | 0.133 | 0.263 | 0.113 | 0.204 |
| **Cell_VS** (%DW) | *End stress* | **39.47** | **34.62** | 0.062 | 0.100 | 0.303 | 0.423 | **39.21** | **27.60** | | 0.026 | 0.069 | 0.176 | 0.297 |
|  | *Harvest* | **36.17** | **37.52** | 0.102 | 0.111 | 0.429 | 0.480 | **35.92** | **33.94** | | 0.069 | 0.117 | 0.387 | 0.611 |
| **Hemi_VS** (%DW) | *End stress* | **26.96** | **25.52** | 0.034 | 0.037 | 0.115 | 0.119 | **24.85** | **24.29** | | 0.041 | 0.031 | 0.170 | 0.125 |
|  | *Harvest* | **25.69** | **25.81** | 0.094 | 0.085 | 0.303 | 0.273 | **25.26** | **24.81** | | 0.059 | 0.059 | 0.248 | 0.245 |
| **SS** (mg.g^-1^ DW) | *End stress* | **143.21** | **219.75** | 0.259 | 0.252 | 4.629 | 6.910 | **265.9** | **287.0** | | 0.246 | 0.141 | 10.88 | 6.744 |
|  | *Harvest* | **236.62** | **258.82** | 0.186 | 0.292 | 5.501 | 9.452 | **252.9** | **269.9** | | 0.129 | 0.225 | 5.449 | 10.12 |
| **AcBL** (mg.g^-1^ DW) | *End stress* | **109.83** | **95.40** | 0.123 | 0.237 | 1.690 | 2.829 | **53.88** | **56.46** | | 0.285 | 0.202 | 2.564 | 1.904 |
|  | *Harvest* | **130.33** | **129.49** | 0.120 | 0.204 | 1.953 | 3.301 | **137.3** | **125.9** | | 0.102 | 0.230 | 2.342 | 4.827 |
